# Complete Genome and Plasmid Sequences of Seven Isolates of Salmonella enterica subsp. enterica Harboring the *mcr-1* Gene Obtained from Food in China

**DOI:** 10.1128/MRA.00114-19

**Published:** 2019-08-01

**Authors:** Yujie Hu, Scott V. Nguyen, Chang Liu, Wei Wang, Yinping Dong, Séamus Fanning, Fengqin Li

**Affiliations:** a Key Laboratory of Food Safety Risk Assessment, Ministry of Health, China National Center for Food Safety Risk Assessment, Beijing, People’s Republic of China; b Centre for Food Safety, School of Public Health, Physiotherapy and Sports Science, University College Dublin, Belfield, Dublin, Ireland; c Food Science and Engineering College, Beijing University of Agriculture, Beijing, People’s Republic of China; University of Delaware

## Abstract

Seven Salmonella enterica subsp. enterica isolates were identified as carrying the *mcr-1* gene, by using a real-time fluorescence quantitative PCR method, from a total of 2,558 isolates which were cultured from various food origins in China between 2011 and 2016. Few complete genomes of Salmonella strains harboring the *mcr-1* gene have been reported to date, so we report here the complete genome and plasmid sequences of all of these isolates to provide useful references for understanding the prevalence of foodborne Salmonella enterica subsp. enterica isolates carrying *mcr-1*.

## ANNOUNCEMENT

The rise and dissemination of multidrug-resistant (MDR) Enterobacteriaceae, especially carbapenem-resistant Enterobacteriaceae (CRE), with mechanisms such as NDM-1, KPC, and OXA-48/181 in the last few decades have led to urgent challenges in the clinical treatment of MDR or extensively drug-resistant (XDR) pathogen diseases (1, 2). In this case, despite having a side effect of nephrotoxicity, colistin is still considered a last-resort antibiotic in the clinical treatment of serious infections caused by CRE (3). However, mobile colistin resistance (MCR), referring to a plasmid-mediated gene encoding a phosphoethanolamine transferase conferring resistance to colistin, was initially reported in 2015 in China and named *mcr-1* (mobile colistin resistance 1) (4). This gene, originating from different bacterial species from human, animal, and environmental samples, has been reported in more than 30 countries across six continents, dating to at least the 1980s (5). Variants of *mcr-1* and other *mcr* genes have been identified consecutively, with the *mcr-8* gene reported in Klebsiella pneumoniae as the latest one (1). While the presence of *mcr-1*-mediated colistin resistance was predominantly reported among Escherichia coli, K. pneumoniae, and Enterobacter spp. in China, data for Salmonella isolates are lacking, particularly for isolates of food origins (6).

Therefore, 2,558 Salmonella isolates recovered from various kinds of food in China between 2011 and 2016 were screened for the *mcr-1* gene by the real-time fluorescence quantitative PCR method in our laboratory, and seven isolates among them were identified as *mcr-1* positive (7). We present here the complete genome and plasmid sequences of all seven foodborne Salmonella enterica subsp. enterica isolates. These genome sequences will be of great use in providing a genetic basis for *Salmonella* spp. harboring the *mcr-1* gene, as references to aid in comparative genomics applications, as well as for epidemiological studies on outbreak detection and surveillance of Salmonella spp. in the future.

A single colony for each strain was grown overnight on brain heart infusion (BHI) broth at 37°C, and genomic DNA was extracted using the TIANamp bacterial DNA kit (catalog no. DP302; Tiangen Biotech, Beijing, China); this was followed by preparation of a 10-kb library from 5 μg of sheared and concentrated genomic DNA using a 10-kb template library preparation and sequencing procedure with the PacBio template prep kit. Whole-genome sequencing was performed using the single-molecule real-time Pacific Biosciences (SMRT PacBio) RS II platform (Tianjin Biochip Corporation, Tianjin, China). SMRT sequencing was conducted using the C4 sequencing chemistry and P6 polymerase with 1 SMRT cell. SMRT Analysis v2.3.0, available from PacBio, was used to perform demultiplexing, base calling, quality filtering of the raw read sequences, and *de novo* assembly according to the RS Hierarchical Genome Assembly Process (HGAP) workflow v3.0. Subsequently, Consed software v28.0 (http://www.phrap.org/consed/consed.html) was used to manually inspect and trim duplicate ends to generate single, complete, and closed sequences for each chromosome and plasmid. The genomes assembled from the PacBio data were then error corrected using Pilon software (v1.23) with Illumina MiSeq sequencing read data, of which a library was prepared with a NEBNext Ultra DNA library prep kit for Illumina (NEB catalog no. E7370), followed by sonication fragmentation (350-bp insert), and loaded onto the Illumina HiSeq platform with a paired-end (PE) 150-bp sequencing strategy (Novogene, Beijing, China) with the HiSeq X Ten reagent kit v2.5 (Illumina, San Diego, CA). The predicted serotypes and multilocus sequencing types (MLST) were identified using the *Salmonella In Silico* Typing Resource (SISTR; https://lfz.corefacility.ca/sistr-app/). Plasmid replicon types or incompatibility (Inc) groups were determined using the PlasmidFinder 2.0 platform (https://cge.cbs.dtu.dk/services/PlasmidFinder-2.0/).

### Data availability.

The genome and plasmid sequence data of all seven Salmonella enterica subsp. enterica isolates have been deposited in NCBI GenBank under BioProject no. PRJNA498334. Automatic annotation was done using the NCBI Prokaryotic Genome Annotation Pipeline (PGAP). The isolate information, sequencing metrics, and genomic data of the seven Salmonella enterica subsp. enterica strains are listed in Table 1.

**TABLE 1:** Chromosome and plasmid sequence accession numbers and additional information for seven Salmonella enterica subsp. *enterica* strains harboring the *mcr-1* gene

Strain or plasmid name	Chromosome or plasmid	*Salmonella* isolate information				Sequencing metrics			Genomic data					
		Serotype	MLST	Yr	Food source	No. of reads	Mean read length (bp)	Coverage (×)	BioSample accession no.	GenBank accession no.	Size (bp)	G+C content (%)	No. of coding genes, pseudogenes, and RNA genes	Plasmid replicon type (Inc group)^*e*^
CFSA122	Chromosome	Typhimurium	34	2013	Pork	113,771	8,504	127.12	SAMN10279393	CP033226	4,990,577	52.1	4,903, 121, 125	NA
pCFSA122-1^*a*^	Plasmid (complete)									CP033224	181,747	46.7		IncHI2A, IncHI2
pCFSA122-2	Plasmid (complete)									CP033225	6,758	46.2		ColRNAI
CFSA244	Chromosome	Typhimurium^*c*^	34	2014	Pork	55,132	10,327	71.51	SAMN10290237	CP033255	4,957,526	52.1	4,883, 110, 122	NA
pCFSA244-1	Plasmid (complete)									CP033253	149,567	45.6		IncHI2A, IncHI2
pCFSA244-2^*a*^	Plasmid (complete)									CP033254	60,381	42.3		IncI2
CFSA12	Chromosome	Typhimurium^*c*^	34	2014	Pork	91,574	9,412	101.28	SAMN10290244	CP033257	4,991,162	52.1	4,868, 110, 125	NA
pCFSA12^*b*^	Plasmid (complete)									CP033256^*d*^	147,918	45.0		IncHI2A, IncHI2
CFSA1096	Chromosome	London	155	2015	Pork	69,175	10,269	89.86	SAMN10291458	CP033348	4,696,663	52.3	4,637, 145, 117	NA
pCFSA1096^*a*^	Plasmid (complete)									CP033347	297,348	46.7		IncHI2A, IncHI2
CFSA231	Chromosome	Derby	40	2016	Pork	65,092	8,680	68.13	SAMN10291561	CP033350	4,834,516	52.1	4,519, 134, 119	NA
pCFSA231^*a*^	Plasmid (complete)									CP033349	33,309	41.9		IncX4
CFSA629	Chromosome	Typhimurium	34	2016	Egg	54,855	9,083	57.39	SAMN10291586	CP033352	4,999,270	52.1	4,937, 117, 125	NA
pCFSA629^*a*^	Plasmid (complete)									CP033351	210,674	45.2		IncHI2A, IncHI2
CFSA664	Chromosome	Indiana	17	2011	Chicken	88,121	8,319	85.69	SAMN10292850	CP033356	4,733,813	52.1	4,760, 176, 120	NA
pCFSA664-1	Plasmid (complete)									CP033353	255,327	47.9		IncHI2A, IncHI2, IncN, IncQ1
pCFSA664-2	Plasmid (complete)									CP033354	41,696	45.4		IncP-1-like *trfA*
pCFSA664-3^*a*^	Plasmid (complete)									CP033355	61,841	42.4		IncI2

a Contains the *mcr-1* gene.

b A spontaneous *mcr-1* gene deletion was observed on pCFSA12 of *Salmonella enterica* subsp. *enterica* isolate CFSA12.

c Predicted to be a potential monophasic variant of *S.* Typhimurium with a serotype antigenic formula of 4,[5],12:i:−.

d The error-corrected sequence of CP033256 by Pilon with Illumina sequencing read data was identical to the previous version, so the accession number version did not change.

e NA, plasmid replicon typing was not applicable for the chromosome. pCFSA664-2 was not predicted to have an Inc group using PlasmidFinder; however, the annotation contained an IncP-1-like *trfA* replication gene from an also-untypeable plasmid, pYDC107_41 (GenBank accession no. CP025711), which is 97.63% identical with 95% coverage to pCFSA664-2 (8).
